# Development of Simple Sequence Repeat (SSR) Markers of Sesame (*Sesamum indicum*) from a Genome Survey

**DOI:** 10.3390/molecules19045150

**Published:** 2014-04-22

**Authors:** Xin Wei, Linhai Wang, Yanxin Zhang, Xiaoqiong Qi, Xiaoling Wang, Xia Ding, Jing Zhang, Xiurong Zhang

**Affiliations:** Oil Crops Research Institute of the Chinese Academy of Agricultural Sciences, Key Laboratory of Biology and Genetic Improvement of Oil Crops of the Ministry of Agriculture, Wuhan 430062, China

**Keywords:** SSR, genetic diversity, genome survey, sesame

## Abstract

Sesame (*Sesamum indicum*), an important oil crop, is widely grown in tropical and subtropical regions. It provides part of the daily edible oil allowance for almost half of the world’s population. A limited number of co-dominant markers has been developed and applied in sesame genetic diversity and germplasm identity studies. Here we report for the first time a whole genome survey used to develop simple sequence repeat (SSR) markers and to detect the genetic diversity of sesame germplasm. From the initial assembled sesame genome, 23,438 SSRs (≥5 repeats) were identified. The most common repeat motif was dinucleotide with a frequency of 84.24%, followed by 13.53% trinucleotide, 1.65% tetranucleotide, 0.3% pentanucleotide and 0.28% hexanucleotide motifs. From 1500 designed and synthesised primer pairs, 218 polymorphic SSRs were developed and used to screen 31 sesame accessions that from 12 countries. STRUCTURE and phylogenetic analyses indicated that all sesame accessions could be divided into two groups: one mainly from China and another from other countries. Cluster analysis classified Chinese major sesame varieties into three groups. These novel SSR markers are a useful tool for genetic linkage map construction, genetic diversity detection, and marker-assisted selective sesame breeding.

## 1. Introduction

Sesame (*Sesamum indicum* L.) is one of the most commonly grown oil crops and has been suggested as the most ancient oil crop [1]. It provides part of the daily oil and protein needs for almost half of the world’s population. It is also directly consumed directly as a leafy vegetable, sweetmeat, cooking ingredient and snack food. Its high regard by consumers has earned it the poetic label “queen of oilseeds” [2] for its high oil content, high oil quality, and strong resistance to oxidation. Sesame oil content in sesame (up to 64% of dry seed) is much higher than that in other edible oil crops, such as soybean (~20%), rapeseed (~40%) and maize (~8%) [3]. The amount of unsaturated fatty acid in sesame oil (>80%), which is beneficial to human health, is also greater than that found in other main oil crops [4]. Moreover, some strong antioxidant compositions (e.g., sesamol, lignan, sesamin, and sesamolin) are rich in sesame oil and possess many potent pharmacological properties leading to physiological effects such as decreased blood lipids and lowered cholesterol levels [5,6,7].

Several types of molecular markers have been developed and applied to sesame genotyping, such as random amplified polymorphic DNA (RAPD) [8,9], inter-simple sequence repeats (ISSR) [10], amplified fragment length polymorphism (AFLP) [11], sequence-related amplified polymorphisms (SRAP) [12] and expressed sequence tags-SSR (EST-SSR) [13]. However, most studies were carried out using random molecular markers and few co-dominant polymorphic markers were used.

SSR markers are widely used for high-throughput genotyping and map construction as they are advantageous due to high abundance, random distribution within the genome, high polymorphism information content (PIC) and stable co-dominance [14,15,16]. The reproducibility, co-dominance, relative abundance and complete genome coverage of SSR markers have made them one of the most useful tools for detecting genetic diversity, genetic linkage mapping, association mapping and evolution analysis [17]. Genomic SSRs and expressed sequence tag (EST)-SSRs, which are considered complementary to plant genome mapping, have been reported in several primary oil crops, such as rapeseed [18], peanut [19] and soybean [20]. Few EST-SSRs were developed and used to detect genetic diversity for sesame germplasm [13,21,22]. However, their use is limited due to relatively low polymorphism and high possibility of no gene-rich regions in the genome. In contrast, genomic SSRs are highly polymorphic and tend to be widely distributed throughout the genome, resulting in more accurate detection of genetic diversity [23].

An elite cultivar, Zhongzhi13, which was introduced to most major sesame planting areas in China over the last 10 years, was sequenced *de novo* by our group using the Illumina Hiseq2000 platform [24]. After reads filtering, 54.5 Gb of high-quality data were obtained, representing approximately 152.7 folds of the sesame genome. The Zhongzhi 13 draft genome was 274 Mb, covering 76.7% of the genome size, which was derived from a 17-mer depth distribution (357 Mb). In this study, sesame genomic SSRs of sesame were identified and developed by whole genome survey. We mined and validated 218 SSR markers and described their application for understanding the genetic relationship of 31 sesame accessions from worldwide. Thirty-two high polymorphism SSR markers were selected as the core SSR markers and used to detect the relationship among 23 major Chinese sesame varieties. These novel polymorphism SSR markers were effectively applied to genotype and genetic diversity analyses.

## 2. Results and Discussion

### 2.1. Frequency Distribution of Different SSR Markers Types

The assembled scaffolds were searched for the presence of SSRs in the genome using the MISA software, and 23,438 putative SSRs from 196 of 1,036 scaffolds (≥2 kb) were identified. The average distance between SSRs was approximately 11.69 kb in sesame genomic DNA. SSRs in plant genomes have been surveyed in many species, such as *Oryza sativa*, *Arabidopsis thaliana* and *Sorghum bicolor* [25]. Genomic SSR number and frequency were quite different among these sequenced plants. SSR numbers in *O. sativa*, *A. thaliana*, *S. bicolor*, *S. indicum* were 70,531, 15,249, 73,658 and 23,438, respectively; the frequencies of SSR occurrence in these species in each locus were 2.75 kb, 2.39 kb, 5.70 kb and 11.69 kb, respectively. SSR frequency in *A. thaliana* was nearly five times greater than that in *S. indicum*.

**Figure 1:**
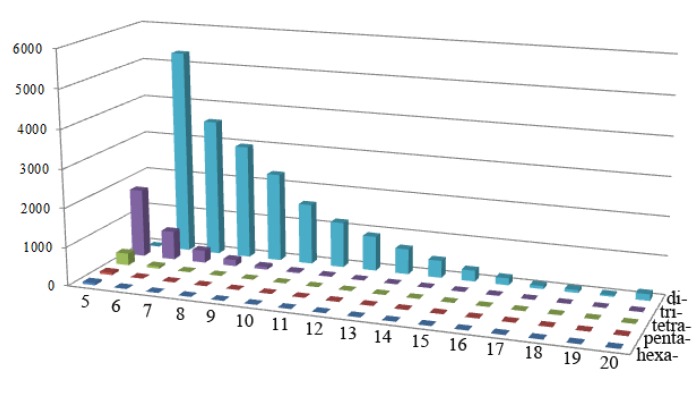
Distribution of various classes of simple repeat motifs with different numbers of repeats in the sesame genome.

SSR loci contained in scaffolds ranged from 1 to 1,049 (average of 119), and almost all scaffolds (192) contained more than one SSR. Among the derived SSR repeats, the dinucleotide motif was present in all scaffolds: trinucleotide, tetranucleotide, pentanucleotide and hexanucleotide motifs existed in 155 (79.08%), 110 (56.12%), 48 (24.49%), and 42 (21.43%) scaffolds, respectively. Short tandem repeats categorised by their unit sizes and the number of repeats are summarized in Figure 1. Dinucleotide repeats (84.24%) comprise the largest group of repeat motifs, accounting for more than three-quarters of the total SSR content. Less commonly found repeats were trinucleotide repeats (13.53%), followed by tetranucleotide repeats (1.65%) and pentanucleotide repeats (0.30%), with hexanucleotide repeats being the least abundant at merely 0.28% (Figure 1). A large proportion of both dinucleotides and trinucleotides was observed, while the rest amounted to less than 2.3%. Although the frequency was diverse, the overall pattern of SSR motifs of particular lengths was similar. Dinucleotide repeats dominated over other types of repeats in all the species and SSR frequency decreased stepwise with increased motif length. Dinucleotide repeats were found to be more than 50% in all genomes. While the dinucleotide and trinucleotide repeats mostly contributed to the major proportion of SSRs, a very small share was contributed by tetranucleotide, pentanucleotide and hexanucleotide repeats. Because high throughput sequencing mainly acquires short read lengths during sequencing, the presence of repeats with large unit lengths might have been decreased.

**Figure 2:**
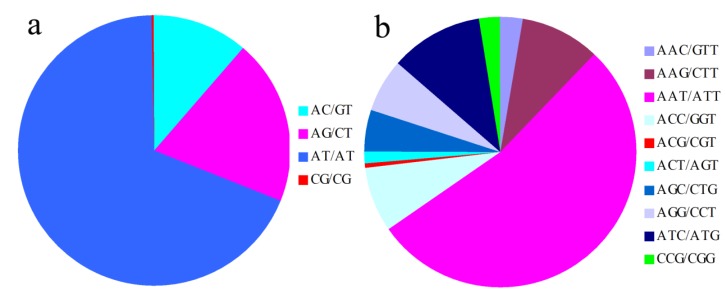
(**a**) Percentage of different dinucleotide repeat motifs in the sesame genome. and (**b**) Percentage of different motifs in trinucleotide repeat motifs in the sesame genome.

SSR motifs consisted of 102 types. Among the dinucleotide repeat motifs, the AT repeat was the most common, representing 68.80%, followed by 19.60% AG/CT repeats (Figure 2a). The predominant trinucleotide motifs (AAT/ATT, ATG/ATC and AAG/CTT) repeats accounted for 59.22%, 11.31% and 9.36%, respectively (Figure 2b). Among the dinucleotide repeat motifs, (AT)n, which was up to 68.80%, was the most common repeat unit in *S. indicum*, (AT)n is also the most common repeat in *A. thaliana*, *S. bicolor*, and *Brassica napus* [25,26].

### 2.2. SSR Marker Polymorphism

We first designed and synthesized 1,500 SSR primer pairs from those scaffolds greater than 2 kb. The majority of SSR loci were dinucleotide repeats (1,097, 73.13%), and trinucleotide repeats (255, 17.00%). Initially, four representative cultivars (Aizhima, Mishuozhima, Gaozhima and Silengzhima) were selected to detect the effectiveness of these primer pairs in through denaturing polyacrylamide gel electrophoresis (PAGE). The four landraces included major sesame characteristics, such as ramiform, rhabdus, black, white, dwarf and tall individuals. Finally, 1,449 (96.60%) SSR loci were amplified successfully in all four accessions, and 218 (14.53%) of them were identified as polymorphic loci in the four germplasm resources.

The frequency of each motif of the 218 polymorphic SSRs is presented in Table S1. Only 13 motif types were included. Most of them were dinucleotide repeats (87.61%), and trinucleotide, tetranucleotide, pentanucleotide, hexanucleotide repeats comprised 9.17%, 2.29%, 0.46% and 0.46%, respectively. Dinucleotide loci in the polymorphism group were greater than that in the genome, indicating that dinucleotide loci had higher diversity than other loci. Among the dinucleotide and trinucleotide repeat motifs, the AT and AAT/ATT repeats, which were up to 79.58% and 65.00%, respectively, were still the most common types.

Subsequently, 31 worldwide accessions of sesame, including 27 landraces and four elites, were used to detect SSR loci polymorphisms. Among these accessions, those from China and abroad were 16 and 15, respectively. The number of alleles (*N_A_*) per locus varied widely among the markers (Table S2) and ranged from 2 to 13, with an average of 5.46 alleles. The frequency of major alleles (*M_AF_*) per locus was 0.1290–0.9677, with an average of 0.4305. In addition, the *H_O_* values were 0.0000–1.0000 with an average of 0.2496, and the *H_E_* values were 0.0635–0.9138 with an average of 0.6853. Lastly, polymorphic index content values were 0.0605–0.8901, with an average of 0.6315. The polymerase chain reaction (PCR) product size ranged from 106 to 280 bp. All the primers produced amplicons in agreement with the expected sizes. Most of the SSR primers (216 primer pairs) showed significantly deviated from Hardy-Weinberg equilibrium (HWE: *p* < 0.05).

Since traditional massive-scale DNA sequencing is relatively high-cost, low-throughput and time-consuming, the development of SSR markers was not fast and limited SSR markers have been developed. Based on mass sequence sesame data obtained by RNA-Seq, 40, 32 and 59 polymorphic EST-SSRs were successfully developed, respectively [21,22,27]. Considering three recently published sets of EST-SSR markers derived from transcriptome studies, available polymorphic SSR and EST-SSR markers are still less than 150. Although EST-SSRs are useful for genetic analysis, their disadvantages of relatively low polymorphisms and high concentration in gene-rich regions of the genome may limit their use [28]. At present, using high-throughput next-generation sequencing, the whole sesame genome was sequenced and SSRs in the genome were detected. For the first time, 1,500 SSR primer pairs were designed from the sesame genome survey and 1,449 (96.60%) primer pairs were effectively amplified. Furthermore, 218 polymorphic SSRs were developed and characterized, of which 137 markers with a PIC value of >0.60 were highly informative.

### 2.3. Genetic Relationship Analysis

**Figure 3:**
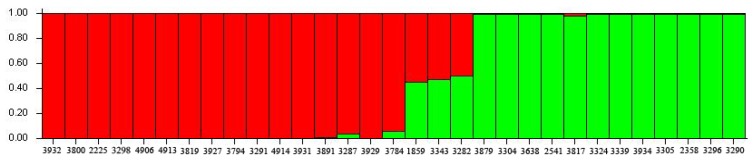
Population structure of sesame accessions. STRUCTURE was constructed by all loci. *K* = 2. Clusters are indicated by different colours. Samples included in all clusters are listed in Table S3.

**Figure 4:**
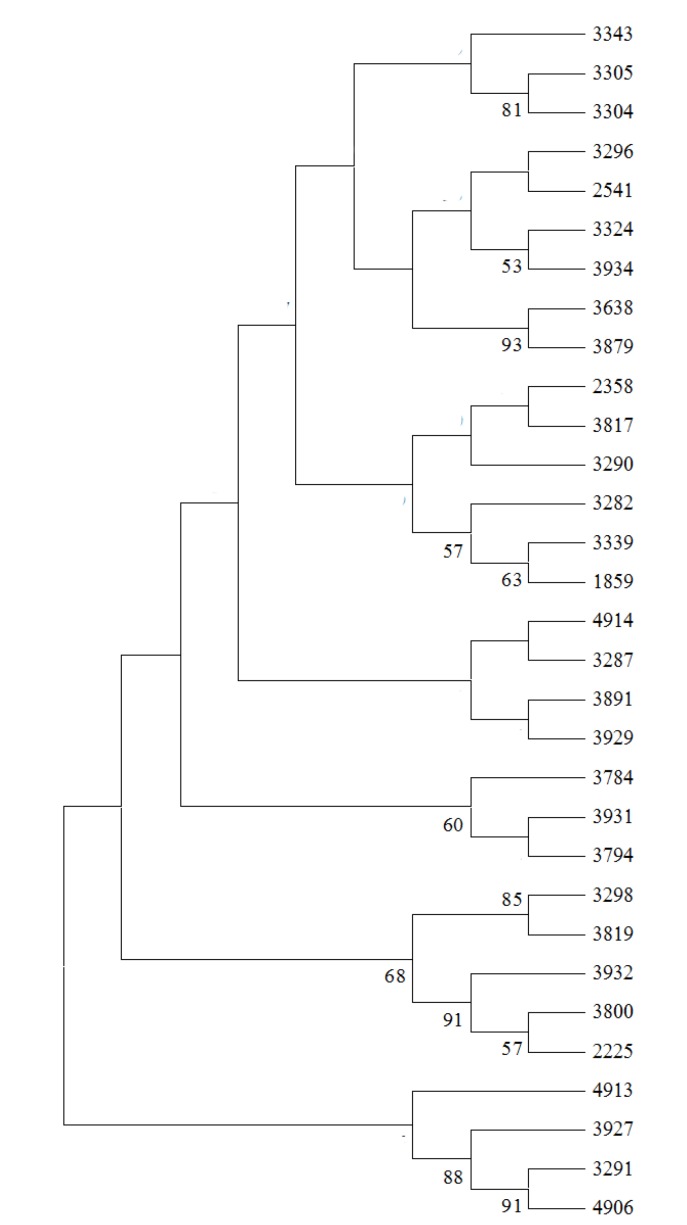
Phylogenetic tree of 31 sesame accessions derived from NJ analysis based on 218 SSR markers. Bootstrap values >50 are shown.

Based on the 218 polymorphic SSRs, we constructed a STRUCTURE analysis to classify the 31 sesame accessions (Figure 3). As *K* values increased from 1 to 10, the LnP (D) value directly increased; meanwhile, the ΔK decreased at *K* = 2 (Figure S1). Thus, STRUCTURE analysis divided all accessions into two clusters. The first group, which is shown in red, included 16 accessions, with five from China, eight from East and South Asia and three from Africa. The second group included 15 accessions, 12 of which were from China and the other three accessions were all from the United States, which were induced mutations from some Chinese sesame germplasm (Table S3). Thus, the 31 sesame accessions could be divided into two groups, and Chinese sesame might have a distant relationship with that from other countries. Phylogenetic trees were constructed for the 31 sesame accessions by neighbor-joining (NJ) analysis (Figure 4) and unweighted pair group method with arithmetic mean (UPGMA) analysis (Figure S2). These sesame samples were also divided into two groups: one was mainly from China and the other was from abroad. The result was in line with the conclusion of STRUCTURE analysis.

Some samples from China and other countries had not been strictly separated in the STRUCTURE and phylogenetic analyses, which may have been due to sample out-crossing. Sesame is mainly self-pollinated, although outcrossing between 5% and 60% has been reported [29]. The sesame samples from other countries and from China were planted together and a few sesame samples might have crossed with each other. However, most samples from China and other countries were still clustered into different groups in the analysis.

Linkage disequilibrium (LD) is the correlation between polymorphisms that is caused by their shared history of mutationand recombination. It plays a central role in association analysis [30]. The allele frequency correlation (*r*^2^) between segregation sites was used to measure the degree of LD. The level of *r*^2^ for all sesame SSR loci was showed in Figure S3. The extent of LD was low for most SSR loci, indicating that LD between SSRs in sesame was weak. Among the 23653 loci pairs, only 1006 (4.25%) of loci pairs were in significant LD at the comparison-wise 0.05 level. This value was less than that in maize [31]. What’s more, many LD blocks were observed in Figure S3, inferring high LD between some SSR loci.

Genetic diversity of sesame has been demonstrated using universal markers [8,9,10,11,12,13]. However, few co-dominant polymorphic markers, ranging from 10 to 50, were used in these studies [27,32]. Few polymorphic markers might not distinguish the samples accurately. In the present study, 218 novel genomic SSRs were developed and used in the genetic diversity analysis. Thirty-one worldwide represent sesame accessions, including both landrace and elite, were collected and used to detect the genetic diversity and relationships. Population structure and phylogenetic analyses indicated that sesame landraces from China and other countries might be inconsistent and elites from China had several common ancient varieties. This was the first attempt at sesame genetic and genotypic diversity analysis using SSR markers developed from de novo sequencing.

### 2.4. Core SSR Marker Development

Among the 218 polymorphic SSR markers, 137 were be considered as high polymorphic SSR markers (PIC > 0.6), while only 102 were easy to identify, repeatable and highly polymorphic. Then, 32 SSR markers were randomly selected from the 102 SSR markers to construct phylogenetic tree of the 31 sesame samples. Finally, we obtained 32 SSR markers to build a UPGMA tree similar to that constructed by the 218 SSR markers (Figure S4). These 32 SSR markers were suggested to be core sesame SSR markers for genetic diversity detection. All of these markers are indicated in Table S2. The core SSR markers were evenly distributed in 16 linkage groups (LGs) of the sesame genome, including two markers in each LG.

**Figure 5:**
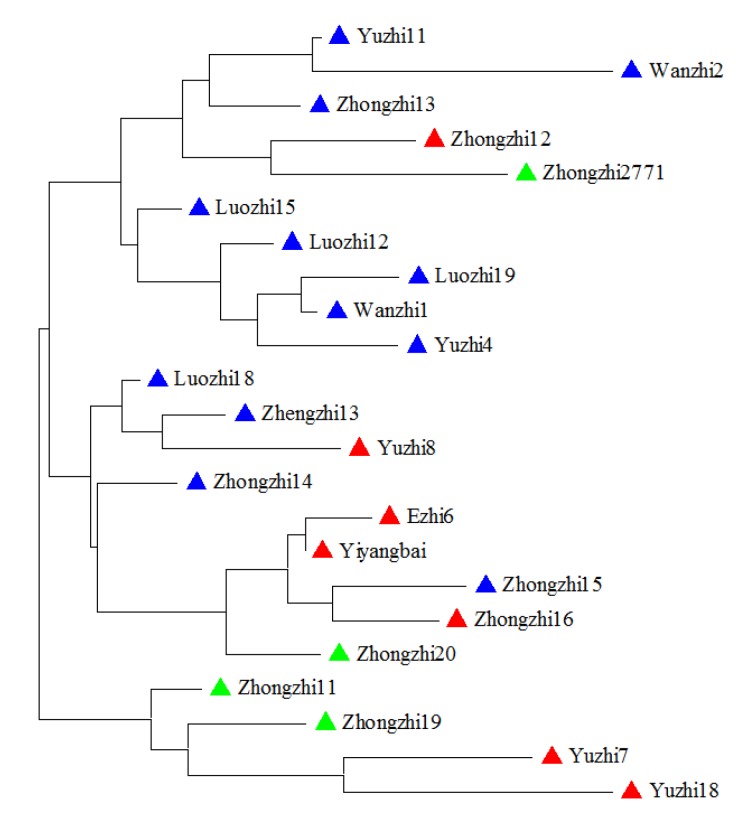
Dendrogram of 23 sesame varieties based on 20 high polymorphism markers. Samples are listed in Table S4.

Twenty additional sesame elites which widely planted in central China were selected. With the three sesame varieties in the 31 sesame samples, we collected 23 elites (Table S4). These elites could be divided into three groups since they had three different major parent varieties (Yiyangbai, Yuzhi4 and Zhongzhi11). To determine if the core SSR markers could reveal sesame varieties genealogy, core SSR markers were used to screen the 23 sesame varieties and construct a phylogenetic tree. Figure 5 shows the relationship among these accessions. Although the varieties were not strictly clustered into three groups, all progenitors tended to be in nearby clades. The results indicated that the 32 core SSR markers could be a useful tool for genetic diversity detection of sesame varieties.

Many SSR markers are typically used for the genetic diversity detection. However, the process is time-consuming for a large number of samples. Core SSR markers have been developed and utilized in some crops, such as soybean [33], maize [34], cotton [35] and rapeseed [36]. Core SSR markers are a set of highly polymorphic SSR markers which selected from plenty genomic SSRs and maximally reflect the diversity and relationship among varieties or germplasm. However, sesame core SSR marker development has not been reported. In this study, 32 core SSR markers were selected from 218 polymorphic SSR markers and could highly reflect sesame variety genealogy.

## 3. Experimental

### 3.1. Plant Materials, Genome Sequencing, and Genome Survey

The sample used for whole-genome de novo sequencing was “Zhongzhi13”, an elite sesame cultivar which was introduced to most of the major sesame planting areas over the last 10 years. In total, eight paired-end sequencing libraries with insert sizes of approximately 180 bp, 500 bp, 800 bp, 2 kb, 5 kb, 10 kb and 20 kb were constructed and sequenced using the Illumina Hiseq 2000 platform.

Thirty-one accessions of sesame collected from worldwide (Table S3), were used to evaluate the SSR suitability for genetic distance analysis. Young leaves were collected and frozen in liquid nitrogen prior to genomic DNA extraction using CTAB methods [37]. DNA concentrations were measured spectrophotometrically at 260 nm, and the extracts were electrophoresed on 1% agarose to confirm the quality. The purified DNAs was standardized at 40 ng/μL and stored at −40 °C.

### 3.2. SSR Identification and Primer Design

The Perl script MIcroSAtelitte (MISA) was used to identify microsatellites in sesame genomes [38]. To identify the presence of SSRs, only two to six nucleotide motifs were considered, and the minimum repeat unit was defined as six reiterations for dinucleotides, and five reiterations for other repeat units. Mononucleotide repeats and complex SSR types were excluded from the study since distinguishing genuine mononucleotide repeats from polyadenylation products and single nucleotide stretch errors generated by sequencing was difficult.

The SSR primers were designed using BatchPrimer3 interface modules [39]. We selected 1.500 primers that met the following parameters: 100–300 final product length (optimal 200 bp), primer size from 19 to 27 bp (optimal 23 bp) and GC content 40%–70% (optimal 50%); the annealing temperature was set at 50–60 °C (optimal 55 °C).

Primers were synthesized by Invitrogen Trading (Shanghai) Co., Ltd. We primarily tested four cultivars (Aizhima, Mishuozhima, Gaozhima and Silengzhima) for 1.500 SSR loci by PAGE to confirm their suitability.

### 3.3. PCR and Gel Electrophoresis

Each 20 μL reaction mixture contained 10× PCR buffer (plus Mg^2+^), 0.2 mM of each dNTP, 5 pmol of each reverse, 4 pmol of the tail primer, 1 pmol of the forward primer, 0.5 units of rTaq polymerase (TaKaRa, Dalian, China) and 40 ng genomic DNA template. Each primer pair had a 20-bp interval according to the expected amplicon size.

DNA amplification was accomplished in a PCR system (Mastercycler: Eppendorf, Hamburg, Germany) programmed at 94 °C for 5 min for initial denaturation, then 35 cycles at 94 °C (30 s)/55 °C (30 s)/72 °C (30 s), followed by the extension step for 10 min at 72 °C. Each PCR product was run on 1% agarose gel at 110 V for a quality check. Subsequently, the PCR products were run on 6% denaturing polyacrylamide gel at 80 W constant gain and polymorphism was detected by silver staining [40].

### 3.4. Data Analysis

The number of alleles (*N_A_*), observed heterozygosity (*Ho*) and expected heterozygosity (*He*) were calculated using POPGENE version 1.32 [41]. Major allele frequency (*M_AF_*), chi-square test for HWE allele frequencies (*P_HW_*) and PIC were calculated using PowerMarker version 3.25 [42]. Genetic similarity among the 31 sesame accessions was calculated according to Dice’s coefficients using Nei’s coefficient index with the FreeTree 0.9.1.50 software [43], and phylogenetic trees were constructed using the NJ and UPGMA method with 1000 bootstrap test [44]. A Bayesian clustering analysis of the samples was constructed by Structure version 2.2 [45]. Five runs of STRUCTURE were carried out for each population number (*K*: from 1 to 10), and each run started with 10,000 burn-ins, followed by 100,000 iterations. LD level of SSR loci in sesame was tested by Tassel 3.0 [46]. Thirty-two high polymorphism SSR markers were selected as core SSR markers. Twenty-three Chinese sesame varieties were screened using these core SSR markers. The Chinese sesame variety dendrograms were constructed by genetic distance.

## 4. Conclusions

Based on the first sequenced and assembled sesame genome, genomic SSR markers were surveyed in the present study. Genome survey identified a total of 23,438 SSR loci from the sesame genome. However, sesame genome SSR frequency was less than that of *Arabidopsis*, rice and sorghum. Different motif frequencies decreased stepwise with increased motif length, and dinucleotide repeats dominated the SSR types. Among the dinucleotide repeat motifs, (AT)n was the most common repeat unit. One thousand five hundred SSR primer pairs were designed and synthesized, and 218 of them were identified as polymorphic markers. Polymorphic SSR markers were used to investigate genetic diversity among 31 worldwide sesame accessions. The population structure and NJ analyses separated all samples into two clusters that corresponded to the geographical areas from which they originated. Thirty-two SSR markers were selected as the core SSR markers and successfully used in genealogy analysis of 23 elites. These SSR markers could provide a useful tool for genetic linkage map construction, genetic diversity detection and marker-assisted selection breeding of sesame.

## Supplementary Materials

Supplementary materials can be accessed at: http://www.mdpi.com/1420-3049/19/4/5150/s1.
