# Supplementary material for: Development of Simple Sequence Repeat (SSR) Markers of Sesame (Sesamum indicum) from a Genome Survey
Source: Molecules. 2014 Apr 22;19(4):5150–62. doi: 10.3390/molecules19045150 (PMC6270694; doi:10.3390/molecules19045150)
Supplement: Supplementary file 1 [file molecules-19-05150-s001.pdf]

Supplementary Files

Figure S1. Estimated Δ*K* values for a given *K* in STRUCTURE analysis.

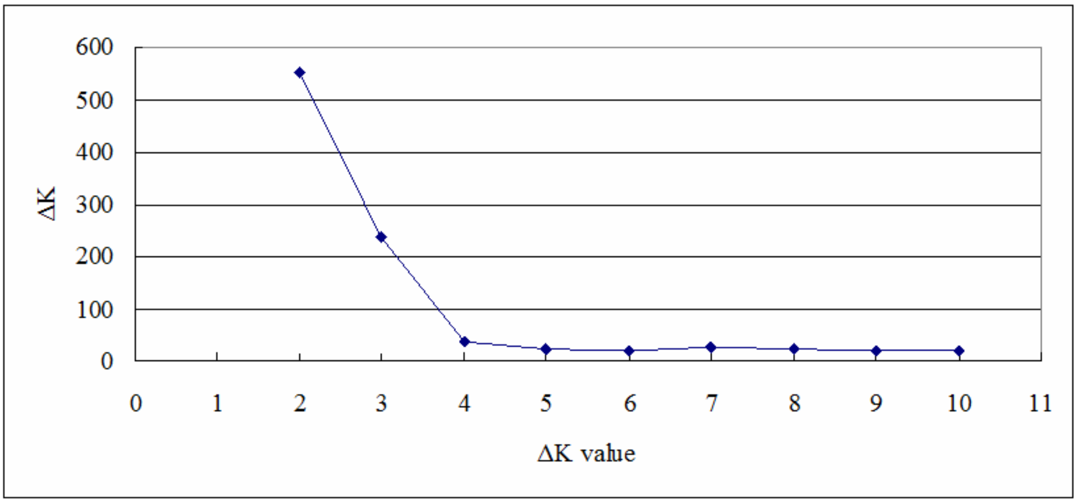

Figure S2. Dendrogram for 31 sesame accessions derived from UPGMA cluster analysis based on 218 SSR markers.

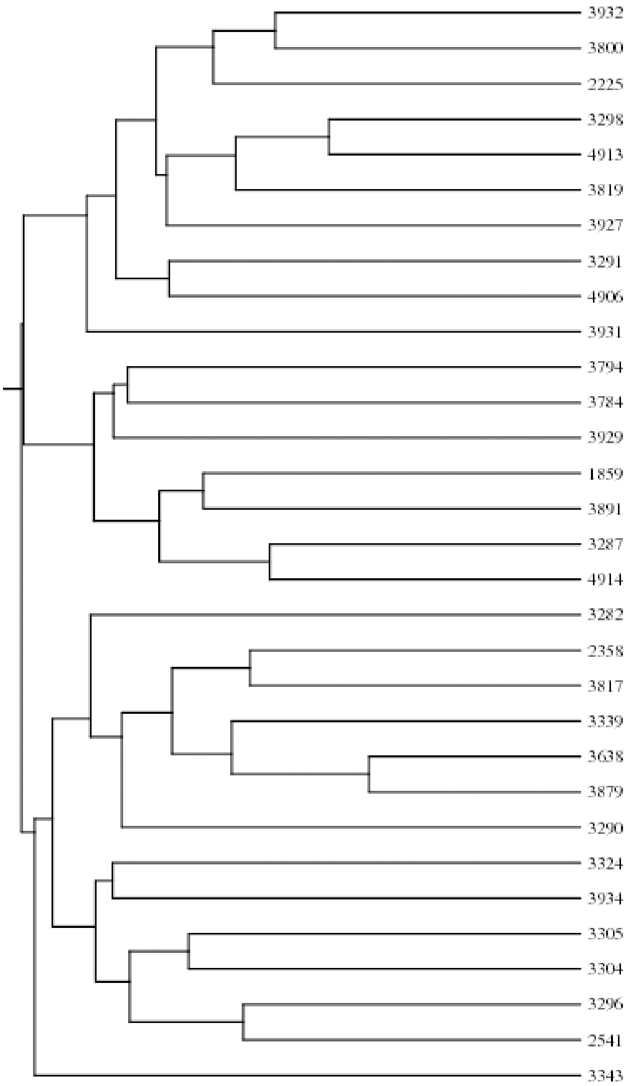

Figure S3. Linkage disequilibrium (LD) patterns for SSR loci in sesame. LD are shown by the r^2 statistic, with white for $r^2 = 0$, grey scale for $0 < r^2 < 1$, and black for $r^2 = 1$.

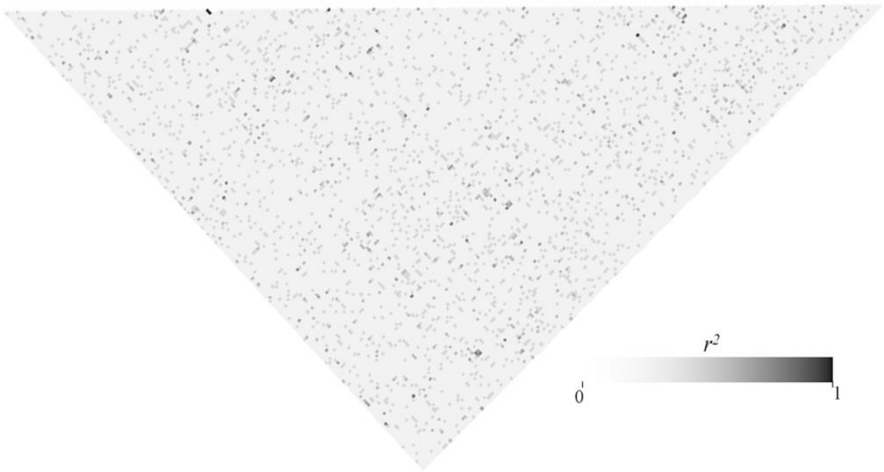

Figure S4. Dendrogram for 31 sesame accessions derived from UPGMA cluster analysis based on 32 core SSR markers.

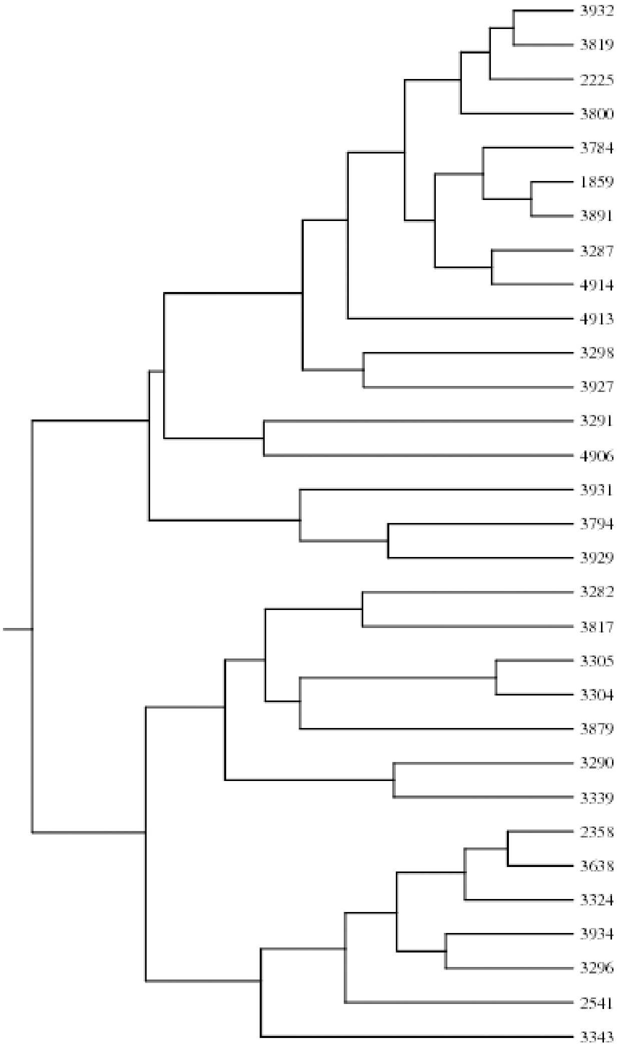

Table S1. Occurrence of the polymorphism SSRs.

SSR Motif	Number of repeats																Total
	**5**	**6**	**7**	**8**	**9**	**10**	**11**	**12**	**13**	**14**	**15**	**16**	**17**	**18**	**19**	**≥20**	
AC/GT	0	3	2	2	2	0	2	0	0	0	0	1	0	0	0	0	12
AG/CT	0	1	1	1	4	0	3	5	2	0	1	0	2	1	0	6	27
AT/AT	0	8	7	4	13	15	30	19	21	13	12	4	1	5	0	0	152
Dimeric (total)	0	12	10	7	19	15	35	24	23	13	13	5	3	6	0	6	191
AAC/GTT	1	0	0	0	0	0	0	0	0	0	0	0	0	0	0	0	1
AAG/CTT	2	0	0	0	0	0	0	0	0	0	0	0	0	0	0	0	2
AAT/ATT	8	2	1	2	0	0	0	0	0	0	0	0	0	0	0	0	13
ACT/AGT	2	0	0	1	0	0	0	0	0	0	0	0	0	0	0	0	3
AGC/CTG	1	0	0	0	0	0	0	0	0	0	0	0	0	0	0	0	1
Trimeric (total)	14	2	1	3	0	0	0	0	0	0	0	0	0	0	0	0	20
AAAC/GTTT	1	0	0	0	0	0	0	0	0	0	0	0	0	0	0	0	1
AAAT/ATTT	2	0	0	0	0	0	0	0	0	0	0	0	0	0	0	0	2
AATT/AATT	2	0	0	0	0	0	0	0	0	0	0	0	0	0	0	0	2
Tetrameric (total)	5	0	0	0	0	0	0	0	0	0	0	0	0	0	0	0	5
AAAAG/CTTTT	1	0	0	0	0	0	0	0	0	0	0	0	0	0	0	0	1
Pentameric (total)	1	0	0	0	0	0	0	0	0	0	0	0	0	0	0	0	1
AGCGGG/CCCGCT	1	0	0	0	0	0	0	0	0	0	0	0	0	0	0	0	1
Hexameric (total)	1	0	0	0	0	0	0	0	0	0	0	0	0	0	0	0	1
Total	21	14	11	10	19	15	35	24	23	13	13	5	3	6	0	6	218

Table S2. Characteristics of 218 SSR markers in this study.

Locus	Genbank NO.	Repeat	Foward primer seq	Reverse primer seq	Tm (℃)	Product size (bp)	Na	M_{AF}	Ho	He	PIC	P_{HW}
SIM001	KJ607657	(AG)9	CTGAAGTTCAAGCTCCTGCC	CCTGACTCCAACACCAACCT	55	249	4	0.7097	0.0000	0.4696	0.4268	0.0000
SIM002	KJ607658	(TAAA)5	TTTTATGGTGTTGTGGGCTG	CAACTTCTGCAACCCATTGA	55	250	6	0.4839	0.0645	0.6769	0.6164	0.0000
SIM003	KJ607659	(TG)7	AGCTATGCCCTCTACCATAGG	CCATTTTGAGGACAATGCAA	55	243	4	0.3226	0.0000	0.7403	0.6773	0.0000
SIM004 *	KJ607660	(AT)6	TTTCCATAAGGGGGTATTTGC	GGGATCGATGGTCTAAAAA TTC	55	245	9	0.2903	0.0645	0.8588	0.8289	0.0000
SIM005	KJ607661	(TA)14	TGGAATGGTCAAATGGGTTT	CTGCCTCATCGTTACACCCT	55	179	10	0.2581	0.0000	0.8778	0.8503	0.0000
SIM006	KJ607662	(TA)12	ACATGTGCTAACGTGGACGA	TAGAAAAACGCAAGCAACCC	55	203	5	0.4516	0.0000	0.7171	0.6638	0.0000
SIM007	KJ607663	(TA)15	AGATGCATTCCCTGGACATT	TTTGGTATGCAAGGATGCAA	55	269	8	0.2581	0.0323	0.8276	0.7892	0.0000
SIM008 *	KJ607664	(AT)11	TCGAGCTGAAGATCGACAAG	TTGCTTTTTGGTTGTTGTGG	55	279	5	0.4839	0.0000	0.6832	0.6244	0.0000
SIM009	KJ607665	(CT)21	GGACTCATTCAGTGGACCGT	TTCTTCCCACCTTCAACACA	55	199	9	0.2903	0.0000	0.8123	0.7718	0.0000
SIM010	KJ607666	(AT)11	TTGCAAAATTTCAAAGAACGTA	TCAAACGAAAGGAGCGAGTT	55	274	5	0.5000	1.0000	0.6235	0.5424	0.0000
SIM011	KJ607667	(AT)8	ATATGGCGGTGTCCTAACCA	CATGCAGAAATAACATGCCA	55	106	4	0.7903	0.0323	0.3665	0.3419	0.0000
SIM012 *	KJ607668	(AC)16	TCCCATCATTCAATTGGACA	GCATGCAAAGACGTGACAAC	55	107	8	0.3387	0.0323	0.7684	0.7181	0.0000
SIM013	KJ607669	(TA)10	TCCTCCATCATGCACTTGAA	TTTCATCCGTTCTCGTCTCC	55	191	2	0.9355	0.0000	0.1227	0.1134	0.0010
SIM014 *	KJ607670	(AT)11	CAACGTAAATTTTGTATTTT ACCAAT	CAAACGACCCCATACTCTTTG	55	272	6	0.2419	0.9677	0.825	0.7841	0.0000
SIM015	KJ607671	(AG)12	TATTAAGGGAGAAGGGGCGT	ATTCCCAAGTCCAAAAAGGG	55	255	5	0.2903	0.0000	0.7805	0.7289	0.0000
SIM016	KJ607672	(AT)15	TTTTTGGGAAGAAATTGCA TAAA	CCCTTTGGGATGGCTACATA	55	263	7	0.2581	0.1290	0.8176	0.7756	0.0000
SIM017	KJ607673	(AT)12	ATGAAACCTAACCAGGGGGA	GAGCTGTAGGTGGTTCAGGC	55	151	7	0.2419	0.0323	0.8361	0.7986	0.0000
SIM018	KJ607674	(AG)9	CGTTTACACCCCCTTCAAAA	CCTCTCATTTTAACATTTTTC GTG	55	274	4	0.2903	0.0000	0.7509	0.6899	0.0000
SIM019	KJ607675	(TC)21	GACCCACATCGAATGTGAAA	TGAACTCTTCAACTATACAATG GGA	55	278	7	0.3226	0.0000	0.7996	0.7572	0.0000
SIM020	KJ607676	(AT)8	GACCAACTCCACATACACTTGC	AAACACCCACCACCCTATCA	55	273	2	0.9677	0.0000	0.0635	0.0605	0.0230
SIM021	KJ607677	(TA)6	AGGCATTAGCACAGTCAGCA	ATGAGACGAGCATCCAGACA	55	251	5	0.4032	1.0000	0.6917	0.6240	0.0000
SIM022	KJ607678	(TC)20	CACCACCACTAAGACAGCGA	TGTGCAGATCATGATAGGGG	55	273	10	0.2581	0.0000	0.8609	0.8298	0.0000

Table S2. *Cont.*

Locus	Genbank NO.	Repeat	Foward primer seq	Reverse primer seq	Tm (℃)	Product size (bp)	Na	M_{AF}	Ho	He	PIC	P_{HW}
SIM023	KJ607679	(AT)11	CACCGCACCGTATAACTTCC	CGTGAATTAAACATTAGTATCC CACA	55	264	6	0.4839	0.0000	0.7086	0.6621	0.0000
SIM024	KJ607680	(TCA)5	GGCCAACCCTTTTCAGATTT	GGGCTTCACAACACAAGACA	55	205	4	0.5000	1.0000	0.6467	0.5736	0.0000
SIM025	KJ607681	(CT)13	TATGTCGGCCGTCGTATCTT	ACATTCTTCCCTAGCTCGCA	55	243	11	0.1290	0.0000	0.9138	0.8901	0.0000
SIM026	KJ607682	(ATA)5	GGGACGTGATGGATATTTTGA	TACCTCCGCCGCTAGCTACT	55	208	3	0.5161	0.9677	0.6134	0.5288	0.0000
SIM027	KJ607683	(ATT)8	CGAAGAATCAAACACAAACCA	CTTGGGCTGTTCTGTTGGAG	55	279	4	0.4355	1.0000	0.643	0.5603	0.0000
SIM028 *	KJ607684	(AT)9	GGCACCAAGTTTTGATGGTT	TTTTGAGATGGTTGAATGGG	55	278	7	0.2903	0.0000	0.8207	0.7812	0.0000
SIM029	KJ607685	(AT)12	ATGATGATGGGGCGAGATTA	CCCACCTACACACACATTGC	55	215	5	0.5484	0.0000	0.6473	0.5968	0.0000
SIM030	KJ607686	(TA)15	TGATCCCTTCAAATTGGCTC	GAAGGCCAATTCAAATCCAA	55	257	6	0.5000	0.9355	0.679	0.6240	0.0000
SIM031	KJ607687	(AT)14	AATTGGACTCCGGCTAGGAT	CGCCCTCATCCTTACAATCT	55	204	5	0.4194	0.0000	0.7234	0.6649	0.0000
SIM032	KJ607688	(AT)9	TGTAATGTATCTTTCAAGGGC AAA	CGTAAAAATCTCGACCGCAT	55	265	4	0.6452	0.0000	0.5373	0.4813	0.0000
SIM033	KJ607689	(AAT)4	GGGCCTATCCCAAGTATTGA	GCTTGCGTCCCAAAAATAAA	55	278	6	0.4194	1.0000	0.7229	0.6664	0.0000
SIM034 *	KJ607690	(TA)13	TAAGGAGGCCACTTGCTCAT	AGTGTTGGGTGAGGGAAAAA	55	259	5	0.3871	0.0000	0.715	0.6502	0.0000
SIM035	KJ607691	(AT)6	TCTTATCCCAACATTCGCAA	TGTCAAGTTGTTTGCAGTTGG	55	133	3	0.4194	0.0000	0.6473	0.5597	0.0000
SIM036	KJ607692	(TAT)8	TGGGTTTTGATTTTGACTTTTTG	CAATGTAAGATGATAGCTAAA TCAATG	55	273	4	0.5000	1.0000	0.5902	0.4938	0.0000
SIM037 *	KJ607693	(AT)10	TGGTTGTTTGATGATTTTCGAC	TCCCACCAAAACCAGGTAAA	55	274	5	0.4516	0.0000	0.7192	0.6669	0.0000
SIM038 *	KJ607694	(TC)25	TATGAACCCAACTTGCACCA	CATTTTGTCTCCTTTCTTTTTA AGG	55	207	9	0.3065	0.0323	0.816	0.7776	0.0000
SIM039 *	KJ607695	(AT)12	GAAAACAGGCACTACCTTCCA	AGTCGGACCAAGTTTGGCTA	55	275	8	0.3710	0.0323	0.8075	0.7723	0.0000
SIM040	KJ607696	(AT)15	CAAGTCGCCATCACACTCAT	TCGAGTTGGAATGCAACAAA	55	241	8	0.3065	0.9677	0.8424	0.8096	0.0000
SIM041	KJ607697	(AT)16	CATCAATCACATGGCACGAT	ATGCGAAATCTTTGCAGCTT	55	268	6	0.3871	0.0323	0.7102	0.6468	0.0000
SIM042	KJ607698	(CA)8	GCATCATCTTGACGCTTTGT	TTGGTTTCTTCGTTTGACCC	55	201	2	0.9032	0.0000	0.1777	0.1595	0.0000
SIM043	KJ607699	(TA)9	GGCGAAAAGAGAAAATGCAA	GGACATAATGGGTATTTTG AGAAA	55	241	7	0.3710	1.0000	0.7573	0.7080	0.0000

Table S2. *Cont.*

Locus	Genbank NO.	Repeat	Foward primer seq	Reverse primer seq	Tm (℃)	Product size (bp)	Na	M_{AF}	Ho	He	PIC	P_{HW}
SIM044	KJ607700	(AT)11	CCTAGAGTGGATGGCATGTG	TTCTGCAGCATCCTCTAACTG	55	247	8	0.3226	0.0000	0.7848	0.7389	0.0000
SIM045	KJ607701	(TA)12	AGCCAGCTGGAATGCACTTA	GCATTTTAGGAGTCGTGGGT	55	150	4	0.4839	1.0000	0.5399	0.4211	0.0000
SIM046	KJ607702	(AT)11	TTGCAAAATTTCAAAGAACGTA	TCAAACGAAAAGGAGCGAGT	55	278	2	0.8710	0.0000	0.2285	0.1995	0.0000
SIM047	KJ607703	(TA)12	TGTCCGAGTGAGGTATGCAA	TTAACTAAGGAGTTTACAGGG AAAAA	55	194	6	0.5000	1.0000	0.605	0.5163	0.0000
SIM048	KJ607704	(TA)9	TGGAGTAGCGAGCATAGAGGA	GGCGATAATACAACCACCAAA	55	280	3	0.5161	0.0000	0.5838	0.4868	0.0000
SIM049	KJ607705	(TA)14	TGGCTTCATTTTGGATGGTT	GAGGAGTGACCTATTTGTTAGA CGA	55	[illegible]7	6	0.4194	0.0323	0.7176	0.6596	0.0000
SIM050	KJ607706	(CA)11	TGGGTTTATTTCAATGCCAA	CGCCAAATTCATCTTGGACT	55	258	4	0.4032	0.9677	0.7107	0.6452	0.0000
SIM051	KJ607707	(ATTA)5	CCATGCATGTAATTAGAGC AAAC	CGCATAAACAATGGTGGCTA	55	245	4	0.2903	0.9677	0.7589	0.6993	0.0000
SIM052 *	KJ607708	(AT)10	CTCGATTCTGGACGGATGAT	AGCGGGTGTTTGATTTGAAG	55	226	4	0.2742	1.0000	0.761	0.7017	0.0000
SIM053	KJ607709	(TA)6	TCGACGCTATTCTTTCACAAG	AAAAGGGGTTGAGTGTCGAA	55	232	9	0.2258	0.0968	0.8768	0.8479	0.0000
SIM054	KJ607710	(TA)11	AGAGCAAATGTTTTGACCGT	CAATGTTCATGTCAATGACGAA	55	249	6	0.4516	0.0000	0.7425	0.7006	0.0000
SIM055	KJ607711	(CT)11	CCTTCAAGCTTGCACCATTT	GCAAACCTATGCATCTCCCT	55	219	3	0.4194	0.0000	0.6642	0.5796	0.0000
SIM056	KJ607712	(TAT)5	GGCACGAACACCTAACTGGT	AAAGAGCCTTTTGTAATTCA CCC	55	122	8	0.2419	1.0000	0.817	0.7747	0.0000
SIM057 *	KJ607713	(TATT)5	TTGCTTGTTGTAAATAAATT GCC	TCGTTATTTGTGAAAGTTTTC TTTTC	55	112	6	0.2903	0.0000	0.7975	0.7515	0.0000
SIM058	KJ607714	(AT)6	TTTTCAGCTTATTTTCACCAATA AA	TCAAGTAATCTTCAACTACTAC CAACA	55	229	3	0.5484	0.0000	0.605	0.5276	0.0000
SIM059	KJ607715	(AT)16	AGATTGCCTGAGAAGCCATT	AATCAATCGCCGTACCTGTC	55	221	9	0.2581	0.0000	0.8694	0.8401	0.0000
SIM060	KJ607716	(AG)15	GGAACACATTGATGCCCTCT	CCACTTGCTCACTCACTCCA	55	276	7	0.2903	1.0000	0.7927	0.7458	0.0000
SIM061 *	KJ607717	(AT)13	TACTGGAACTTGGTACGCCC	GCATAACCAAACATCTAGT GGC	55	252	5	0.3226	0.0000	0.7594	0.7038	0.0000
SIM062	KJ607718	(TC)9	GGGGTGGAGTGTAATTATCCG	GTCATGCTCCAAAAATGGCT	55	269	9	0.2581	1.0000	0.8361	0.7999	0.0000
SIM063	KJ607719	(AT)12	CTTGTTGTGGTCTTGGACCC	GGACAGCAAGAGCATGATGA	55	270	7	0.2581	0.0000	0.8334	0.7957	0.0000

Table S2. *Cont.*

Locus	Genbank NO.	Repeat	Foward primer seq	Reverse primer seq	Tm (℃)	Product size (bp)	Na	M_{AF}	Ho	He	PIC	P_{HW}
SIM064	KJ607720	(TA)10	CTGTGTGGTCATTCAGTGGG	AACATCCCTCATATTTGTTTGACA	55	240	4	0.5161	0.0000	0.6473	0.5810	0.0000
SIM065	KJ607721	(TAT)4	CAAAAGATCATGGGTTTGGG	ATGTCGCGCAATCAATAGGT	55	193	3	0.5484	0.0000	0.5732	0.4803	0.0000
SIM066	KJ607722	(AT)13	AACCATTGAGAAAGGGACGA	TTATACGGAGGAGTGACGGC	55	204	3	0.5000	1.0000	0.5653	0.4577	0.0000
SIM067	KJ607723	(TA)13	GCACTCCACTTTGGGTAAGC	TACCAAGGATCGGCCTTATG	55	246	8	0.2258	0.0000	0.8567	0.8235	0.0000
SIM068	KJ607724	(AT)10	GACGCACTGAATCAAATCAAA	GAGGAGAGGTGCCATGACTT	55	238	5	0.3226	0.0000	0.77	0.7169	0.0000
SIM069	KJ607725	(AAAAG)5	CGAAGGGTGTGTATGTGGTG	GGAAGGACGTAATTGGTATTTG	55	278	5	0.4839	0.0000	0.6832	0.6244	0.0000
SIM070	KJ607726	(AT)14	ACGCGAAGGCTCAAGTAGAA	TCGGAATTTTGGATTTGGTC	55	247	4	0.4516	0.0000	0.6854	0.6169	0.0000
SIM071	KJ607727	(CAAA)5	GTTGTCTGCTGTTTCGAACG	TCATCCATACATCCAATGTACAAA	55	216	4	0.5000	1.0000	0.6118	0.5251	0.0000
SIM072	KJ607728	(AT)9	CGCTTGAATTAATTGCATCTACC	CCAAGTGAACATAGAAATCTGCC	55	226	2	0.6129	0.0000	0.4823	0.3619	0.0000
SIM073	KJ607729	(AT)11	TCGAACAAGCACAGGAATTG	GGGCGTAAATGGTATTTTGAGA	55	277	6	0.5000	0.9677	0.606	0.5182	0.0000
SIM074	KJ607730	(AT)14	GCACCCTTAACTTGGCATTC	TTGATGCAGTTATGTGCTCACT	55	149	9	0.2581	0.0000	0.8525	0.8195	0.0000
SIM075 *	KJ607731	(AT)12	CCCCTCTCAAATAAGCCCTC	AGGAAGGAGGGTGTCCCTAA	55	196	11	0.3548	0.0000	0.8186	0.7851	0.0000
SIM076 *	KJ607732	(TA)9	GAAATCGAAGCATTTTGGGA	TGAATCCAAGTTCACGATCAA	55	133	13	0.2903	0.0645	0.88	0.8552	0.0000
SIM077	KJ607733	(CAT)8	TGCCAAAAACACAAGAATGA	TGCATGCTCTCGTACGTGTT	55	258	6	0.2581	0.0000	0.8102	0.7658	0.0000
SIM078	KJ607734	(AG)9	CCAGACCCAAACCCAATAGA	TGCATTTAAGGCTGTGCAAC	55	264	7	0.2581	0.0968	0.8292	0.7905	0.0000
SIM079	KJ607735	(TG)9	AATTTATCCGCCTGCACAAC	CGACATTGCATAGATGAGCG	55	154	11	0.2097	1.0000	0.8942	0.8683	0.0000
SIM080	KJ607736	(TA)9	TGAAAAGTGCCATGTCAGCA	GGGCTACCGATTGGATTTCT	55	239	4	0.5000	1.0000	0.6319	0.5544	0.0000
SIM081	KJ607737	(TC)21	TTCCTCAAGATTAGCTGCGG	CCGAGCACCTTAAACCAAAC	55	268	2	0.9032	0.0000	0.1777	0.1595	0.0000
SIM082	KJ607738	(TA)10	ATTCTTGAACTGGGTCGGGT	GGGAAAAACAATTGTGTATATTTGC	55	206	2	0.9355	0.0000	0.1227	0.1134	0.0000
SIM083	KJ607739	(AT)8	TCTTCCGATTAATATTTTCGTGC	TCATGTATTGATTGAATCGACG	55	210	3	0.8710	0.0000	0.2348	0.2152	0.0000
SIM084	KJ607740	(TA)6	TGTCCTGGAACACTAAGGGC	CCAGTTCCACTGTCCCTCAT	55	232	3	0.5000	1.0000	0.6245	0.5401	0.0000

Table S2. *Cont.*

Locus	Genbank NO.	Repeat	Foward primer seq	Reverse primer seq	Tm (℃)	Product size (bp)	Na	M_{AF}	Ho	He	PIC	P_{HW}
SIM085	KJ607741	(AAG)5	ACTGATTTTGTAGCAGGGCG	TGTCTCCCAATTATCACGCA	55	267	2	0.5645	0.0968	0.4997	0.3708	0.0000
SIM086	KJ607742	(TA)18	TCAGTACCTTAAATCAAGCCGA	GTGGGCGCAGTTAGCTGTA	55	249	8	0.2581	0.0645	0.8567	0.8246	0.0000
SIM087	KJ607743	(AT)13	TATTGGGTATCGTCACGGGT	TTCTAAGGGATTTGGTTAGCCT	55	218	6	0.4516	0.0000	0.734	0.6884	0.0000
SIM088	KJ607744	(AG)7	TTTAGGAAGGATGTGATGGG	GGTGTGTGTTGTGTGCTGTG	55	262	2	0.7258	0.0323	0.4045	0.3188	0.0000
SIM089	KJ607745	(TA)7	CCATTTTTCTTCCAAAGGGA	TCTTATCCCAACATTTAATTTA	55	259	8	0.4516	0.9355	0.7504	0.7124	0.0000
SIM090	KJ607746	(GT)11	ATGATGGTGATGCTGATGGA	TGAAATCTGTGGGAGAAGCC	55	253	4	0.3871	0.0000	0.7192	0.6535	0.0000
SIM091	KJ607747	(ATG)5	CCCCTGACCTTTCAGTTACG	ATCCTATGCACCACACAGCA	55	199	8	0.1935	1.0000	0.8757	0.8458	0.0000
SIM092	KJ607748	(TA)7	ATTTCCAGCCAAATTGACCA	TTGATGCCAACATTATCGGA	55	244	2	0.6774	0.2581	0.4442	0.3415	0.0400
SIM093	KJ607749	(TA)14	CGACCAATGATATTTTTGCTTGT	TGGAAATGTTGGGAATGTGA	55	208	2	0.8065	0.0645	0.3173	0.2634	0.0000
SIM094 *	KJ607750	(CTCCGC)5	TGCTCGATCCAATATCACCA	TCTCACCTCTTCTCCCTCTCC	55	225	6	0.2903	0.0000	0.7975	0.7515	0.0000
SIM095	KJ607751	(CTG)5	ACTGGGCAATGGTTTTCTTG	GGGATGAAATGAGGACAGGA	55	204	3	0.5161	0.0000	0.6134	0.5288	0.0000
SIM096 *	KJ607752	(TA)13	CTCATGTGGAACGAGGCATA	ATGGCCACCACCTAACATTC	55	227	4	0.4194	0.0000	0.698	0.6310	0.0000
SIM097	KJ607753	(TA)13	TCCAAATACGCAACGAACAA	TTCATCCATCCATCCAAAGA	55	279	5	0.4516	0.1613	0.7155	0.6610	0.0000
SIM098 *	KJ607754	(AT)15	CTTTGATTGGGCCACCCTA	TGTTTGTTCTTCTTCCCCCA	55	280	6	0.3387	0.1935	0.7673	0.7152	0.0000
SIM099	KJ607755	(TC)6	AGATCGTAACTCCGGACCCT	TTTGGAGGAGGAAATGTTGC	55	259	3	0.7419	0.0000	0.404	0.3401	0.0000
SIM100	KJ607756	(AT)7	TCAGTCAAGTCAGGCCAGTG	ACGGGCATAATCAACCCATA	55	176	2	0.8710	0.0000	0.2285	0.1995	0.0000
SIM101	KJ607757	(AAT)5	TTTAGAAAGGGCGTAATGGG	AATTGTTTGTGTGCGCATGT	55	237	6	0.4677	1.0000	0.7054	0.6522	0.0000
SIM102	KJ607758	(AAT)6	GCCCAATTAACCCATGTTGT	CAAGAGAGAGAAAGTGATGGG TTT	55	279	3	0.5000	1.0000	0.5241	0.3983	0.0000
SIM103	KJ607759	(TA)7	CGCTTTTTAGCCGTTGAGAC	TGCCGATCCTTCTCCTTCTA	55	161	4	0.6452	0.0000	0.5415	0.4892	0.0000
SIM104	KJ607760	(AT)11	GAGACACCCAACGATTTTGC	TCCCTTCGAAATGGTATCTCC	55	251	2	0.5806	0.0000	0.495	0.3684	0.0000
SIM105	KJ607761	(AT)14	CGGCCGAGAGAGTGAGACTA	CGTAATTGAGCGCATTGAAA	55	265	4	0.5161	0.0000	0.6388	0.5689	0.0000
SIM106	KJ607762	(AT)15	TTCACAAGGGGGTAATTTGC	TCTGGCATTGCAAACACCTA	55	162	5	0.2581	0.0000	0.8017	0.7547	0.0000
SIM107	KJ607763	(AT)13	CCTTGCCAATGAAACTTTCTC	AATCATTTCCAGTGGGGACA	55	229	8	0.2258	0.0000	0.8546	0.8209	0.0000
SIM108	KJ607764	(AT)10	TGCAAATGTTTGGATAGCACA	TGAGAATCGGCCAATTAAGAA	55	280	10	0.2097	0.6774	0.8789	0.8500	0.0000
SIM109	KJ607765	(AT)11	CTGCCATTGCATCATCAACT	GGCGAGGTAGGCACAGATTA	55	253	8	0.2581	0.7742	0.8186	0.7772	0.0110
SIM110	KJ607766	(TA)13	CGCACTGGAGTGAGAGAGTG	CATCATCTCGAACGCTTGAA	55	234	5	0.4194	0.0000	0.734	0.6800	0.0000

Table S2. *Cont.*

Locus	Genbank NO.	Repeat	Foward primer seq	Reverse primer seq	Tm (℃)	Product size (bp)	Na	M_{AF}	Ho	He	PIC	P_{HW}
SIM111	KJ607767	(AT)7	TTTGCCTCTTAACATTTTCCA	TTGGGTCTGTCTCCTCGTTC	55	269	3	0.6774	0.0000	0.4781	0.4048	0.0000
SIM112	KJ607768	(TA)7	TTGGAGCCAGGAAGATTTTG	ATGGCCCAGGTTAGTGTCTG	55	260	3	0.8065	0.0000	0.3342	0.3016	0.0000
SIM113	KJ607769	(GA)12	GCTGATGAAATCTCGACCGT	GTTGCTTGCATTTTTCCCTC	55	223	4	0.7742	0.0000	0.3871	0.3569	0.0000
SIM114	KJ607770	(AT)6	TATCACGCGACCGAAATACA	ATCGGGGGTTGTGTTAGTCA	55	244	8	0.2742	0.0323	0.8382	0.8024	0.0000
SIM115	KJ607771	(AT)14	CGTAGTGGTTCCCCTCACAT	ATGCTTTCCCCCAAATAACC	55	179	3	0.5806	0.0000	0.5711	0.4910	0.0000
SIM116 *	KJ607772	(TTA)5	AATTGTCCTTCTCGGTGGTG	CTGCATCAGGATCTCCGAA	55	270	7	0.3548	1.0000	0.7456	0.6919	0.0000
SIM117	KJ607773	(TA)12	AAGGGTGGGAGGAGAGAGAA	AACGGTTTGGACAAAGATCG	55	235	6	0.2258	0.0000	0.8186	0.7758	0.0000
SIM118	KJ607774	(AT)10	CCCGAAAATAGGATTTCTAACA	TTTTGGACTGCTATTGAGGGA	55	184	6	0.5484	0.0000	0.6663	0.6279	0.0000
SIM119 *	KJ607775	(AT)12	CAGCATTAGCGTCTCGACAA	TTTTTCATCCACCTAGTCCGA	55	251	7	0.2258	0.0000	0.8334	0.7950	0.0000
SIM120	KJ607776	(AT)11	GCGTTGTAATAACAGGGCGA	TGATTGGTTTGATTCGGTCA	55	232	6	0.3871	0.7742	0.761	0.7116	0.0040
SIM121	KJ607777	(AT)9	GTGCATGGATGGAATGACAA	TGCACCTCACAAAACCAACT	55	252	6	0.3710	0.9677	0.734	0.6773	0.0000
SIM122	KJ607778	(AT)12	TTAAGGTGGCTGATTTTGGG	GAATAGTCCGGCAATGCTAAA	55	234	5	0.2581	0.0000	0.7953	0.7470	0.0000
SIM123	KJ607779	(AT)11	AAGCAATGCTGCTGCTACAA	CCCCTACCCCAAACCTAAAC	55	207	10	0.2258	0.0000	0.8694	0.8393	0.0000
SIM124	KJ607780	(GA)12	TGGCACCATGATAGGACAAA	AAACTTGATCACCCACAAAAC	55	247	5	0.3226	0.0000	0.7678	0.7152	0.0000
SIM125	KJ607781	(TC)8	TCTACATTTACACGTGCCGC	AAAATG GCACAGTCTTCGCT	55	278	5	0.4194	0.9677	0.7097	0.6457	0.0000
SIM126	KJ607782	(AT)14	TGTCCGTGTTTCAGCTTTTCT	CAAATTGCGGGCTCATCTAT	55	265	7	0.2258	0.0000	0.8334	0.7950	0.0000
SIM127	KJ607783	(GTT)5	AAGGAAGCGAAGAGAATGGG	GCGTGGTTTCTTGAACGATAA	55	278	8	0.2097	0.9355	0.8599	0.8270	0.0180
SIM128	KJ607784	(TA)13	TGATTTTATTGTGGGCTTTCC	CTTGCATGCAGACACGATG	55	277	6	0.4194	0.0000	0.7403	0.6903	0.0000
SIM129	KJ607785	(GT)6	CGGATTAAATTGGAGTTCGC	GTACCTCTGTTTCCTCCGCA	55	268	4	0.4839	1.0000	0.5812	0.4792	0.0000
SIM130	KJ607786	(AT)7	TGCTGCAAGATTACAAGCAAA	TAGCTGCCAGCAATTCATAA	55	148	3	0.5000	1.0000	0.5389	0.4198	0.0000
SIM131	KJ607787	(TA)18	TCACTGAAGGCGAAAAGAAAA	AAATCAGAAAGGACGTATGGG	55	280	3	0.8710	0.0000	0.2348	0.2152	0.0000
SIM132	KJ607788	(TCT)5	GGCAAAGTCGTTCCAAAAAT	AACTTACCTGGTGGTGGCTG	55	258	3	0.8226	0.0323	0.3125	0.2859	0.0000
SIM133	KJ607789	(TA)11	TTCAAACTTCATGGGAGGAGA	GATTGGGCTGAGTCCAGAAA	55	208	3	0.7419	0.0000	0.4209	0.3747	0.0000
SIM134	KJ607790	(AG)12	AACCTCACCGCATTCAACAT	TATCGCTCACACTGTTCGGA	55	153	4	0.5161	0.0000	0.6071	0.5220	0.0000
SIM135	KJ607791	(AAT)6	AACTTGAACAGGATAAATTG GAGC	TGCTGGTGTAATTGTGTCTGTG	55	276	4	0.7097	0.0000	0.4611	0.4103	0.0000

Table S2. *Cont.*

Locus	Genbank NO.	Repeat	Foward primer seq	Reverse primer seq	Tm (℃)	Product size (bp)	Na	M_{AF}	Ho	He	PIC	P_{HW}
SIM136	KJ607792	(AT)18	GATGTCATCAATTTGTTGTTTAA GA	TGCTTGTGGCTAGAAAACT TAGAA	55	265	8	0.2581	0.0000	0.8292	0.7916	0.0000
SIM137	KJ607793	(TA)11	TTATGGGGAGAACTAGGGGC	TGGGGTTTCCATTATTGCTC	55	181	8	0.2903	0.0645	0.8186	0.7788	0.0000
SIM138	KJ607794	(TG)7	GCAAGCATGAATTAAAGTGTCC	ATGTTGATCCACACGGGC	55	229	5	0.7097	0.0000	0.4802	0.4479	0.0000
SIM139 *	KJ607795	(CT)17	GAGAGAGATTTTGAGGCGGA	TGTTGATGGTGTCTGGTATTGA	55	273	7	0.3226	0.0000	0.8313	0.7967	0.0000
SIM140	KJ607796	(TA)13	AGTTATTTTAAAGGGTCTTC TCGTT	AAGAGTCTGCCCATGACACC	55	160	7	0.4516	0.0645	0.7419	0.7001	0.0000
SIM141 *	KJ607797	(TA)14	CACGTAATTCATCGTGATCCA	AGAAGTTTGAGTTTGGCGGA	55	253	7	0.3548	0.0000	0.7827	0.7371	0.0000
SIM142	KJ607798	(AT)12	TGTCCACATCAAATGTCCCT	TGTGGAACTCGAATTTGTGAA	55	213	5	0.2903	0.0000	0.7932	0.7451	0.0000
SIM143	KJ607799	(AT)14	GCAGAAATAGAACTTGGAG GGA	TCTGCTAGAACATCGAAAC CAA	55	189	6	0.1935	0.0000	0.8419	0.8043	0.0000
SIM144 *	KJ607800	(AT)8	AATTACAAGGCGGGTAGCCT	CAACTTCTTCATGTTCGTCCTT	55	261	4	0.4194	0.0000	0.6959	0.6278	0.0000
SIM145 *	KJ607801	(TA)18	ATCCCCTGAGGAGTGTCGAT	GCACAAACATCCCTGACCTT	55	151	8	0.3226	0.0000	0.8271	0.7918	0.0000
SIM146	KJ607802	(AG)13	GCACCACAATTGTCGAAGG	TTCATCTCACAAGTTCCACCA	55	277	7	0.4516	0.0323	0.7335	0.6885	0.0000
SIM147	KJ607803	(AT)12	AAAGCACGAATTGATGCCC	GCAATTAATCGTTTAGTTCCAT ATT	55	278	7	0.2903	0.0000	0.8144	0.7741	0.0000
SIM148	KJ607804	(AT)11	GCTTCCAAGGATACACCCAA	TGACTCTGCCTGTCCTACCA	55	197	5	0.6129	0.0000	0.5817	0.5320	0.0000
SIM149	KJ607805	(AG)11	CGAATCCTGTTCTCCCAAAC	AAACCAAACATGGGGAGTTG	55	187	2	0.6452	0.0000	0.4654	0.3530	0.0000
SIM150	KJ607806	(AT)11	TTATGGTTTGAACTGCCACAA	ACGACGACGACGACAATCTA	55	242	5	0.4839	0.0000	0.6959	0.6431	0.0000
SIM151	KJ607807	(GT)8	TATCCAGGGGAAAACCAGAA	TTGGATTTTCCTTCTCACGC	55	212	3	0.4839	0.0000	0.6346	0.5503	0.0000
SIM152 *	KJ607808	(AT)13	GAGCCAAATCTTAAGGGTTTCA	TTGTGAGGATCGGAAAGACC	55	207	7	0.2903	0.0000	0.8313	0.7943	0.0000
SIM153	KJ607809	(AT)10	TTCCAATTCTACAAGCGCAG	CCGATCAAAACTAGTATGGAA	55	274	3	0.8387	0.0000	0.2834	0.2540	0.0000
SIM154	KJ607810	(AT)13	CGTATGACGTTAACTTCGCAGA	AAAAAGGAGGTGGCGATTAGA	55	187	10	0.2581	0.0000	0.8673	0.8375	0.0000
SIM155	KJ607811	(AT)15	TTTTCTCCTATTGCGCTCGT	GGACCCCAACAACAGCTAAA	55	264	7	0.2581	0.9032	0.844	0.8088	0.0010
SIM156	KJ607812	(TA)9	TTAGGGCCTAGGGTACGGAT	TGCATCACTTTGTGGAGTGG	55	256	5	0.3226	0.0000	0.7636	0.7080	0.0000
SIM157	KJ607813	(AC)6	CACCACCCCATTGGACTAAC	GAGAAGGGGATTTTGCTGTG	55	229	4	0.3871	0.0000	0.7192	0.6535	0.0000
SIM158	KJ607814	(AT)11	TTGTGCATTTTGAAGGATCG	GGCATTGGCAAACTTTGATT	55	184	3	0.5806	0.0000	0.5796	0.5043	0.0000

Table S2. *Cont.*

**Locus**	**Genbank NO.**	**Repeat**	**Foward primer seq**	**Reverse primer seq**	**Tm (℃)**	**Product size (bp)**	**Na**	**M_{AF}**	**Ho**	**He**	**PIC**	**P_{HW}**
SIM159	KJ607815	(AT)10	ACTTGTGGAAATGGTCGGAG	GCGTGGATTAGGTGGTGACT	55	266	4	0.5806	0.0000	0.6092	0.5543	0.0000
SIM160 *	KJ607816	(AT)11	CCATACTGCTGCTGTTGCTG	TCCATCAAGAGTTTCTCGGG	55	271	5	0.5161	0.0000	0.6621	0.6041	0.0000
SIM161	KJ607817	(AT)11	GTGGCGGAGTACAGTTTGGT	TCATTTTCAATGTCACAGGAGG	55	214	7	0.2581	0.0000	0.844	0.8086	0.0000
SIM162	KJ607818	(AT)13	TTACACGATCAACATAAATTCA AGA	TCCATAAGAATTTTTCGCCTTT	55	263	3	0.6129	0.0000	0.5584	0.4903	0.0000
SIM163	KJ607819	(TC)17	GTAACCCTATTCCCCCGTGT	GCCAGAAAACAATCAAGGGA	55	195	8	0.2903	0.0000	0.8292	0.7924	0.0000
SIM164	KJ607820	(AT)16	TCAAACAAAAGTGACAGCCAA	CATGCATGAGTCGTAACCAGA	55	255	6	0.2903	0.0000	0.8038	0.7590	0.0000
SIM165	KJ607821	(AT)9	TGTGTAGTCCCTAGACGGTGAA	TTTATGGTGAACTGACCAAAAT GT	55	228	3	0.5484	0.0000	0.5732	0.4803	0.0000
SIM166	KJ607822	(AT)9	GGACGCATACACAACCACAT	TTTCGATAACCGAGGAATGG	55	280	7	0.2903	0.0000	0.8228	0.7836	0.0000
SIM167	KJ607823	(AT)13	CCTCCCTAGATTTTCGATTGC	GGCACATGTTCACAAATCCA	55	280	7	0.4194	0.0000	0.7573	0.7132	0.0000
SIM168	KJ607824	(AT)12	GGAAATCAGGTCCCTGTCAA	TTCCCTTTCCGATGTACCAA	55	233	4	0.5161	0.0000	0.6388	0.5673	0.0000
SIM193	KJ607849	(TA)16	GCCAAAACAAAGGATTCAAGA	TGAGCTTTGTGTGACCATGA	55	169	6	0.2903	0.1613	0.7927	0.7450	0.0000
SIM194	KJ607850	(AT)11	AGTAATAGGGTCGGCGGATT	AACGTCCGAGTACTTCCTGG	55	237	3	0.6452	0.0000	0.5288	0.4655	0.0000
SIM195	KJ607851	(ATA)7	TGGTCCGATTAACTCATGTTG	CTGCAACCCAAACCTAGGAG	55	210	5	0.3710	0.0323	0.7525	0.6985	0.0000
SIM196	KJ607852	(AT)11	TCACCAACTTCTCATTCCAAAA	GGGTTGTTTGCATTTGTTCC	55	250	4	0.3387	0.9677	0.7356	0.6731	0.0000
SIM197 *	KJ607853	(AT)15	CAATGGATAAGGAGGCGAAA	ATCGGTCATTCCAATCCTCA	55	262	7	0.1613	0.0000	0.8652	0.8326	0.0000
SIM198	KJ607854	(AT)13	CATAAAACCAGCAGAGCAGC	TAAACCTCGACCTGACGCTT	55	250	5	0.3871	0.0000	0.7298	0.6695	0.0000
SIM199	KJ607855	(AT)11	TCCATCGATGCTGGACTGTA	CAAGTTAAATATATGCTGTACA TGTGG	55	246	6	0.2258	1.0000	0.825	0.7838	0.0000
SIM200	KJ607856	(TA)10	CATCAGTGCCTTTGTTGGTG	TCCAATTTTGTTTCCATCCG	55	274	4	0.4032	0.0645	0.6996	0.6291	0.0000
SIM201 *	KJ607857	(TA)13	TCATTAACCCATCATTGCGA	TGCTCACACATAACAGTTGGG	55	248	6	0.3226	0.0000	0.789	0.7417	0.0000
SIM202	KJ607858	(AT)11	ACTTGCAATTTTAGTCCGGC	CCAAACCAAAACGTACCCAC	55	260	6	0.3871	0.0000	0.7171	0.6572	0.0000
SIM203	KJ607859	(AT)11	GGACCAAAAGTTTGACACGG	GGAGGTGAGAAGTCGCGTAG	55	260	6	0.5645	0.6774	0.6399	0.5951	**0.1320**
SIM204	KJ607860	(AAT)5	TTGACTTGCCTCATGCATTT	TGCAGTTCAATTGTCTGGGA	55	253	7	0.2742	1.0000	0.826	0.7863	0.0000
SIM205	KJ607861	(CA)6	ATTATGCGCGTCAGTGTGTG	CGCTATAATATTTGGGGGCA	55	229	4	0.3548	0.9677	0.7171	0.6515	0.0000
SIM206	KJ607862	(TA)9	GAAAACGAAATGGAGGTTTGA	TTAACACAAAGAGCTGCTCAC	55	257	4	0.5806	0.0000	0.6007	0.5404	0.0000

Table S2. *Cont.*

**Locus**	**Genbank NO.**	**Repeat**	**Foward primer seq**	**Reverse primer seq**	**Tm (℃)**	**Product size (bp)**	**Na**	**MAF**	**Ho**	**He**	**PIC**	**PHW**
SIM207	KJ607863	(AT)11	TGTTGTTTGACCGTCTTCCA	TCGGGCTAGAAACCAACAGT	55	267	6	0.2419	0.9355	0.8324	0.7933	0.0000
SIM208	KJ607864	(AATT)5	TTTAATAATTCATTTGATGG ATGG	ACTTCAAGTATGCGTTGGGC	55	276	7	0.3548	0.0323	0.8043	0.7654	0.0000
SIM209	KJ607865	(TA)6	CGTCCGAGGATGTGTGTTTA	GCTCGTTATAATATTTGGGACA	55	273	2	0.7742	0.0000	0.3554	0.2885	0.0000
SIM210	KJ607866	(AT)14	CACGCTAACATTTATATCATTT GACC	CCAGTTTGTGTACTCGATCCC	55	155	4	0.5161	0.0000	0.6388	0.5673	0.0000
SIM211	KJ607867	(AT)11	CTGCTATCCACGTGCTTCAA	GGGGTTTAAATTCAAAATTCCA	55	222	3	0.8387	0.0000	0.2877	0.2639	0.0000
SIM212	KJ607868	(AT)10	AATGGAACATGAAAACCGGA	TCCTTGTCATCCAATCACTCC	55	265	3	0.4516	0.0000	0.6515	0.5671	0.0000
SIM213	KJ607869	(TA)12	CCAACAAGATGGAATGTCCC	AGGACAAGTCAATGGATGAAA	55	263	8	0.2419	1.0000	0.8461	0.8108	0.0000
SIM214	KJ607870	(AT)9	CCCACACGTTAGTGATTCCA	ATTTGCATGCATGAAGCGTA	55	272	4	0.5806	0.0000	0.6113	0.5577	0.0000
SIM215	KJ607871	(AT)15	GCCAGAGGTGGTCACAATTT	GCTTCTCAACTATTCCTCCCTG	55	278	5	0.2903	0.0000	0.7848	0.7347	0.0000
SIM216	KJ607872	(AT)11	CTTGACCTAATTGGGAGGGG	TGGGAGTATTGGGGTCTTCA	55	211	6	0.2581	0.0000	0.8228	0.7822	0.0000
SIM217	KJ607873	(TA)15	ACGTTGTATACATAGATCGC CTC	AATTAATATTGGAGAAAGGCA ATATG	55	269	7	0.3710	0.0968	0.7673	0.7190	0.0000
SIM218	KJ607874	(TA)11	ATAATGCGACCCATCCTCAA	CCCACCGTCTCTAACCAAGA	55	232	9	0.3065	0.9677	0.8001	0.7597	0.0000
Mean							5.4862	0.4305	0.2496	0.6853	0.6315	0.0026

Note: SSR markers are listed according to ascending order in different fluorescent dyes. Shown for each primer pair are the repeat motif, primer sequences, Tm (℃), product size in Zhongzhi13 (bp), number of alleles detected (Na), observed heterozygosity (Ho), major allele frequency (MAF), expected heterozygosity (He), polymorphism information content (PIC) and Chi-square test for Hardy-Weinberg equilibrium (P_{HW}). P_{HW} over 0.05 are in bold. *: Core SSR markers.

Table S3. Details of the 31 sesame accessions used in polymorphism detection.

Code	CGB NO.	Name	Origin	Seed color
1859	ZZM3838	Zhima8131	Shangrao, Jiangxi, China	White
2225	ZZM5418	Zhongzhi 15	Wuhan, Hubei, China	White
2358	ZZM0830	Yiyangbai	Yiyang, Henan, China	White
2541	ZZM2541	Silengzhima	Zhushan, Hubei, China	White
3282	ZZM0795	Zihuaye 23	Shangcai, Henan, China	Yellow
3290	ZZM4186	Baizhima	Qiongzhong, Hainan, China	White
3296	ZZM4728	Zhongzhi13	Wuhan, Hubei, China	White
3298	-	H98	Wuhan, Hubei, China	Black
3324	ZZM2289	Aizhima	Jiayang, Shangdong, China	White
3339	ZZM2748	Mishuozhima	Dongyang, Zhejiang, China	Black
3638	ZZM0146	Jizhi 1	Shijiazhuang, Hebei, China	White
3794	ZZM2831	Bahuama	Wannian, Jiangxi, China	Black
3817	ZZM4790	Xiangheizhi	Xiangyang, Hubei, China	Black
3819	ZZM0902	Fuyangsilengcao	Fuyang, Anhui, China	Brown
3879	ZZM5396	Jinzhi 2	Taiyuan, Shanxi, China	White
3891	ZZM5408	Zhuzhi 18	Zhumadian, Henna, China	White
3934	-	Xiaolima	Dehong, Yunnan, China	Brown
3927	WZM5444	725	Afghanistan	Brown
3304	WZM4247	Youxianxing	America	Black
3305	-	Juanye	America	Black
3343	WZM3081	82Noins	America	White
3932	WZM5449	L161	Egypt	White
4913	WZM1544	K1	Guinea	Brown
3929	WZM5446	847	India	White
3287	WZM4562	342782	India	Yellow
3800	ZZM2831	Shuiyuan 117	Korea	White
4914	WZM3085	Suke 5	Mozambique	White
3291	WZM1523	Miandianhei	Myanmar	Black
3931	WZM5448	CLSU-1	Philippine	Black
4906	WZM1530	41298	United Arab Emirates	Black
3784	-	V6	Viet Nam	White

CGB NO.: China Genebank NO.

Table S4. Details of the 23 sesame varieties used in genetic genealogy detection.

Name	CGB NO.	Origin	Parents	Seed color
Yiyangbai	ZZM0830	Henan, China	-	White
Ezhi6	ZZM4789	Hubei, China	Yiyangbai&Ezhi1	White
Zhongzhi12	ZZM1438	Hubei, China	Yiyangbai&CLSU-9	White
Zhongzhi16	ZZM5419	Hubei, China	Yiyangbai&Yitiaobian	White
Yuzhi7	ZZM3413	Henan, China	Yiyangbai&Zhongzhi7	White
Yuzhi8	ZZM4546	Henan, China	Yiyangbai&YItiaobian	White
Yuzhi18	ZZM5412	Henan, China	Yiyangbai&Yuzhi11	White
Yuzhi4	ZZM3410	Henan, China	Yiyangbai&Zhuzhi1	White
Zhongzhi13	ZZM4728	Hubei, China	Yuzhi4	White
Zhongzhi14	ZZM4729	Hubei, China	Yuzhi4&84-411	White
Zhongzhi15	ZZM5418	Hubei, China	Yuzhi4&Suxianzhima	White
Yuzhi11	ZZM4547	Henan, China	Yuzhi4	White
Zhengzhi13	ZZM5402	Henan, China	Yuzhi4&KKU3	White
Luozhi12	ZZM4593	Henan, China	Yuzhi4&Zheng89H142	White
Luozhi15	ZZM5405	Henan, China	Yuzhi4	White
Luozhi18	ZZM4783	Henan, China	Yuzhi4&Luozhi12	White
Luozhi19	ZZM5406	Henan, China	Yuzhi4&Yuzhi8	White
Wanzhi1	ZZM4785	Anhui, China	Yuzhi4&Buyuxi	White
Wanzhi2	ZZM4786	Anhui, China	Yuzhi4&Xiaozibai	White
Zhongzhi11	WZM4597	Hubei, China	Yuzhi4	White
Zhongzhi19	-	Hubei, China	Zhongzhi11	White
Zhongzhi20	-	Hubei, China	Zhongzhi11&Suxianzhima	White
Zhongzhi2771	ZZM5421	Hubei, China	Zhongzhi11	White

CGB NO.: China Genebank NO.
